# Fecal contamination of urban parks by domestic dogs and tragedy of the commons

**DOI:** 10.1038/s41598-023-30225-7

**Published:** 2023-03-01

**Authors:** Kensuke Mori, Melanie Rock, Gavin McCormack, Stefano Liccioli, Dimitri Giunchi, Danielle Marceau, Emmanuel Stefanakis, Alessandro Massolo

**Affiliations:** 1 Graduate School of Human Development and Environment/Department of Human Environmental Science, Kobe University, Kobe, Hyogo Japan; 2 Department of Community Health Sciences, Cumming School of Medicine, University of Calgary, Calgary, AB Canada; 3 Grasslands National Park, Parks Canada Agency, Val Marie, SK Canada; 4 Ethology Unit, Department of Biology, University of Pisa, Pisa, Italy; 5 Department of Geomatics Engineering, Schulich School of Engineering, University of Calgary, Calgary, AB Canada; 6 Department of Ecosystem and Public Health, Faculty of Veterinary Medicine, University of Calgary, Calgary, AB Canada; 7 UMR CNRS 6249 Chrono-Environnement, Université Bourgogne Franche-Comté, Besancon, France

**Keywords:** Environmental social sciences, Diseases, Risk factors, Animal behaviour

## Abstract

Contamination of public parks by dogs is a potential source of conflict among park users, causing “tragedy of the commons” problems. Besides the social conflict, feces can pose serious health risks to both dogs and humans. In this study we analyzed the extent and patterns of the distribution of dog feces in the urban parks of the City of Calgary. We collected dog feces from randomly selected locations in the urban parks. The average density of dog feces by the different dog leash policies of the parks and the distribution pattern of the fecal density within the parks were assessed, and the total contamination of the public parks for the entire city was estimated. We found off-leash parks to be significantly more contaminated than other types of parks. We estimated 127.23 g/ha of dog feces are left unpicked in city parks in total every week. Dog feces were found more often and in greater amount in off-leash parks, and near park entrances and parking lots, than in on-leash parks and away from the park entrances. These results suggest that public park visitors, especially those visiting off-leash parks, are likely to be exposed to large amounts of dog feces. Designation of parks as on-leash and educating dog-owners may be an effective approach for reducing the fecal contamination.

## Introduction

Urban parks increase the quality of life in large metropolitan areas, providing stress relief and various health benefits for residents^1–3^. Parks also provide dog-owners places for walking their dogs, promoting physical activity^4–6^, social interactions, and sense of community with other park visitors^2,7,8^. However, dogs in urban parks can also cause conflicts^9^. Dogs can deter physical activities by making others feel unsafe^4,8,10^. Allocating public space as dog-parks to avoid user conflicts can results in dissatisfaction among non-dog-owners^2^.

Dog fecal contamination of public parks is particularly concerning. Dog feces act as deterrents for other park users including other dog-owners, and their presence often leads to loss of trust in park managers and local authorities as negligent^1,11,12^. Dog feces can also transmit zoonotic parasites^13,14^ and contaminate water^15^. Fecal contamination of parks could be considered an example of “tragedy of the commons,” a type of environmental dilemma where there are finite resources that are shared by multiple groups, exclusion or regulation of the use of the resources are impossible or difficult, and the use of the resources by one group will reduce the available resources for others^16,17^. City parks can be considered a common resource shared by park users, whose value could be degraded by dog fecal contamination. Strict enforcement of dog-related bylaws is difficult to implement and could diminish the dog owners’ enjoyment of parks. As this tragedy of the commons is of increasingly politicized problem in many cities not just in Canada but across the world^12,18–23^, this should be a matter of grave interest for any urban planners.

The City of Calgary has pet policy that was highly successful in reducing dog bites and dog-related complaints, and is considered a model for other cities^24–26^. Calgary offers incentives to promote licensing of all the pets with city-run programs and services such as veterinary care, and runs educational campaigns for pet owners^26^. As such, several studies have been conducted on the park visitors and dog owners on their use of public parks and their perception in Calgary. McCormack and Rock^27^ studied the relationships between the socio-demographic characteristics of neighborhoods and physical features of small neighborhood parks and found that dog-walking and dog-related activities were some of the most common use of those parks. They then studied the process of changing and implementing an off-leash policy on these public parks in the City of Calgary, and the effects of off-leash policies on the behavior of dog-owners and their use of the public parks, including the resulting fecal contamination were studied^1,28^. These studies had mixed results on the relationships between fecal contamination and the off-leash designation, and had key limitations that further study was needed. One of the limitations was that it only studied small parks. The City of Calgary has several larger parks that are accessed by people from wider area. These large parks are used by many dog-owners in the city and are of great importance in understanding the overall patterns of park use by dog-owners. High prevalence of gastrointestinal and potentially zoonotic parasites in dog feces such as *Giardia*^24^, and at least one case of highly lethal *Echinococcus multilocularis*^29^, added the urgency for assessment of dog fecal contamination in the public parks of the city as a whole.

In this study we followed up on the work by Rock et al.^1^ and focused on dog fecal contamination of urban parks in Calgary (Canada) in relation to their dog leash policy (no dogs, off-leash, on-leash, and mixed), environments, and landscapes. We aimed to A) estimate the quantity of dog fecal contamination in urban parks in a North-American metropolitan area (Calgary, AB, Canada); B) to understand its spatial patterns; and to C) assess the relationships between the amount of fecal contamination, park management in terms of dog leash policy, and environmental factors. Whilst we had no specific expectation on the quantity of dog feces per hectare, we expected to find higher dog fecal contamination in parks with an off-leash policy, and in areas immediately surrounding the park entry points.

## Materials and methods

### Study area

This study took place in the City of Calgary (AB, Canada; 51° 5ʹ N, 114° 5ʹ W), in south-eastern Alberta. The city covered an area of 848 km^2^ and population of 1,090,936 in 2011 at the time of the sampling, and 1,285,711 in the most recent census^30^. Elevation ranges from 965 to 1304 m above sea level. The climate is predominantly cold and dry (with an average annual high temperature of 10.5 °C and low temperature of − 2.4 °C). The city encompassed 75 city parks and natural areas, and one provincial park, where residents walk their dogs. The habitats of those parks were grasslands in dry areas, forests in well-drained areas and willow shrublands in wet soils areas^31^.

The Calgary bylaw requires dog owners to keep their dogs on leash in public areas except on the designated off-leash areas, and to remove their feces promptly^32^. However, these bylaws were not strictly adhered by dog owners^1^. The city is divided into four sectors, with northeast (NE) residents generally in lower income than the other sectors, which influenced the pattern of people’s activity in the parks and the policy-making process for designation of off-leash areas^28^.

### Sample collection

**Figure 1:**
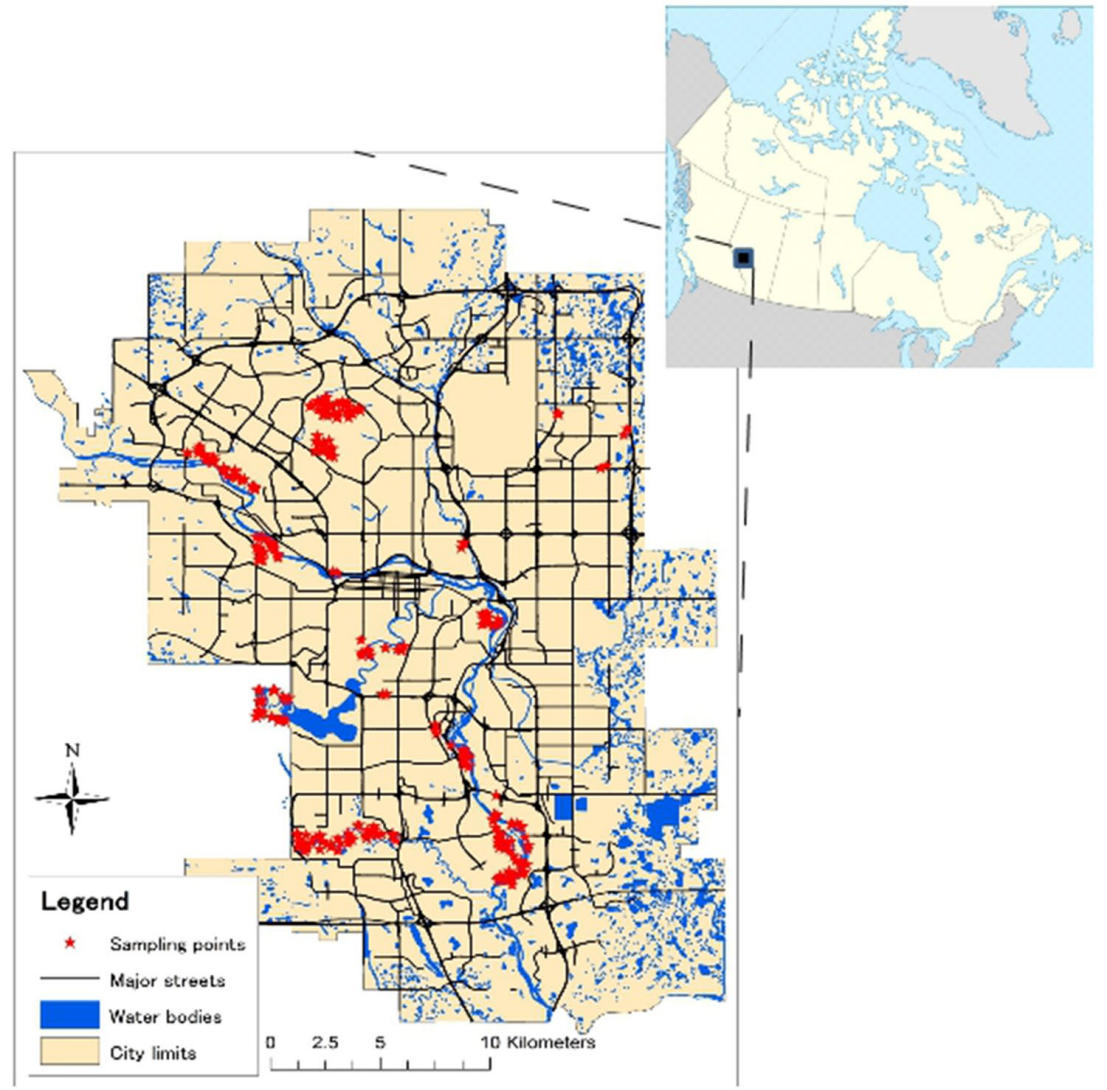
Map showing the City of Calgary, and the locations of sampling points within the dog fecal contamination surveys carried out in 2011 in the city of Calgary (AB, Canada). The map was created with ArcGIS10 (ESRI, CA, USA), using geographic data created by the City of Calgary. The blank map showing territory governed by provinces and territories of Canada was obtained from Wikipedia under CC BY-SA 3.0.

In June and August of 2011 feces of dogs, coyotes and other animals (mostly Canada goose *Branta canadensis* and white-tailed deer *Odocoileus virginianus*) were collected from 16 parks and one bird sanctuary in the city (Fig. 1). The 17 areas were selected to include the four sectors of the city (NW, NE, SW, SE), the main habitat types (grasslands, forests, and shrublands), and the different dog management bylaws. The sampled parks also included two of the largest parks in the city, namely Nosehill Park and Fishcreek Provincial Park, and most of the large-sized parks (> 100 ha) of the city, where a lot of dog-related activities took place. The selected parks ranged in sizes from 1 to 1348 ha. Despite the overall very good socio-economic conditions in Calgary, the four sectors of the city are somehow descriptive of different socio-economic conditions, with the NE sector being the one at lower income. The dog bylaws consist of the following: dogs not permitted (“no dog”); only dogs on leash allowed (“leash on”); dogs allowed without leash (“leash off”); and parks with both “leash on” and “leash off” areas (“mixed”).

Within the 17 areas, we used a random sampling design to identify sites for monitoring fecal contamination: we assigned random points in the sampled parks roughly proportional to their size (15.94 points/km^2^, range 13.22–18.67). Exceptions were made for small parks (Taradale, Martindale, Meadowlark, and West Hillhurst), where two sampling points were assigned regardless of its size. Exceptions were also made for very large parks (Fishcreek Provincial Park and Nosehill Park) due to logistics, where two sub-areas of the parks were designated wherein the sampling points were assigned. Random points were generated using a random point algorithm implemented in ArcGIS10 (ESRI, CA, USA).

Each random point was marked with a 25 cm metal bar in the ground and a sampling plot was defined as the circular area within a 10 m radius surrounding the point. Plots were then visited twice in June and twice in August 2011; the first visit to clear the areas of feces, and the second visit one week later to collect the feces. This was done to assess the weekly rate of fecal deposition. The feces were identified to species by size, shape, content, and texture^33^. In Calgary there were only two canid species (dog and coyote), and in previous studies our group developed significant experience in identifying coyote feces and tested our efficiency with molecular tools^34–36^ and because coyote feces were normally visually distinct from those of dogs due to their content of animal hair, bones, and plant matters, dog feces identification was relatively simple. Each fecal sample was bagged, tagged with a unique identifier, weight, and the species. The feces were then brought to the lab and placed at − 80 °C for 48 h to inactivate any *Echinococcus multilocularis* eggs^37^. The feces were then weighed and kept at − 20 °C.

### Geographical data

The size of each park was obtained from the City Parks website when available or calculated using ArcGIS10 (ESRI) otherwise. Maps of areas managed by the City Parks Services and areas of “off-leash” areas, and the city land cover map (5 m resolution; 2015) were obtained from the City of Calgary, Park Sector office.

The environmental variables characterizing each sampling points of 2011 survey were analyzed using ArcGIS10. To assess the ease of access to the sampling points by dog owners, the distances to the nearest developed areas and trails from the sampling points were calculated. These distances were also associated with the ease of accesses park amenities such as trash bins and dog waste disposals bins, typically located at the park entrances and in parking lots. We defined “developed area” as the land cover types of “roads/railroads” and “buildings/paved roads.” Maps of trails and footpaths inside the parks were also obtained from the City of Calgary. The sampling points were also classified as whether they were within off-leash areas when in a mixed park. Terrain features as slope, aspect, and the ruggedness of the terrain (standard deviation of slope) were calculated using Digital Elevation Model (DEM) with a resolution of 0.75 arc per second (approximately 18 m) developed by Natural Resources Canada in 2012 as a proxy for the terrain variables characterizing each sampling point.

### Data analysis

#### Fecal contamination by leash policy

Although feces of several species were collected, only the samples identified as dog feces were analyzed in this study. The average density of dog fecal contamination was calculated both as the “number of dog feces” and as “mass of dog fecal matter” per week per hectare. The differences in the average densities of contamination in parks with different leash policy classes (no dog, on leash, off leash, and mixed) were tested using one-way ANOVA. Martindale, Meadowlark, Nosecreek, Taradale, and West Hillhurst parks were removed from this analysis because of their small size and small number of sampling points (2 or 3). A Tukey’s honestly significant difference test was used to test the significance of pair-wise differences between the leash policy classes^38^.

Based on the calculated average densities (both in number and mass) of feces in off-leash areas and on-leash areas, the total amount of feces in the parks across the entire city was estimated. We assumed the survey in 2011 would be a representation only of the snow-free period of roughly 6 months, as opposed to the entire year, because we expected different behaviors of dogs and dog owners in winter, and a different detectability of feces in snow. Although not precise, this gives us at least a rough estimate of the total fecal contamination of the urban parks in the City of Calgary during a 6 months snow-free period. The total area of parks in the city was calculated using ArcGIS 10 from the map of the City Parks Services and map of off-leash area. Although the map of the City Parks Services did not distinguish “no-dog” areas from other areas, the “no-dog” areas were small in sizes and therefore we assumed that they could be ignored in our estimate.

#### Feces distribution within parks

The spatial distribution of dog feces within the city parks were modelled by fitting a mixed-effects Poisson distribution model to estimate the number of dog feces found at each survey location (dependent variable) as a linear function of effects due to the dog leash policy at each site (fixed factor), to the environmental characteristics of the plot (fixed factors and continuous variables), after controlling for the clustering of sites within parks (random intercept).

The environmental variables considered were: the accessibility (distance from parking lots and trails), the land cover type of the surveyed plots, and average slope of the area. The land cover of the plots were defined as the land cover type that covered more than half of the plot (> 150 m^2^). Ultimately only the land cover type of “grassland” was used because the correlation of each land cover types with the fecal number, and our observation in the field, suggested natural grassland to be most relevant among the plots we sampled. The slope variable was simplified into categorical variable of being flat or slope, because the resolution of DEM data were too coarse for the plots, and because we suspect that differences in few degrees would not make significant difference in the behavior of dogs or dog owners. The threshold of 10° was chosen. The distance from trail was simplified as a categorical variable of being ”near trail” and “away” from trail, using 50 m as threshold distance. Most of the sites with feces were found within 50 m from trails (see supplementary material Fig. S1). The dog leash policy classes were defined as “off leash,” “on leash” and “mixed.” Because we found zero feces in the “no dog” parks, they were not included in the model. All the models with every combination of the variables were compared using the corrected Akaike Information Criterion (AICc) and the best performing model was selected^39^. The selected model was assessed using likelihood ratio test against the model with only parks as random factor as “null” model.

Analyses were carried out using *glmmTMB* package of R [version 1.1.1; 40]. The assumptions for the mixed-effect Poisson distribution models were assessed using *DHARMa* package of R [version 0.4.3; 41].

Unless otherwise stated, means are reported with their standard errors (SEM). All the statistical analyses were conducted with R software, version 4.1.0^42^.

This study did not handle any human or live animals, and did not require any ethics approval. The research workers were informed of the risks of infection to *Echinococcus multilocularis* and other pathogens while collecting the animal feces. Proper precautions were taken to avoid infections.

## Results

### City park fecal contamination

A total of 53 dog feces was found in 259 random point surveys of 2011. On average, 0.2 dog feces per plot (range: 0–6) was found during the 2 weeks of survey, which translates to 6.51 feces per ha per week. Fecal average weight was 39.06 g (± 2.74). On average 7.99 g of feces per plot (range: 0–313.01) was found, equivalent to 127.23 g per ha per week. If we are to assume that our surveys represent roughly the 6 months (~ 26 weeks) of the snow free times of the year, we estimated a total of 169.26 feces and 3,307.98 g of feces per ha in half a year. The highest density of the number of feces was found in Southland Park at 27.85 feces per ha per week, whereas the highest density in terms of mass of feces was found in West Hillhurst at 1362.37 g per ha per week (Table 1).

**Table 1:** Leash policy, area size, city quadrant, and number/mass of dog feces found in each park during a survey from random points within urban parks in the City of Calgary (AB, Canada) in the year 2011.

Area	Leash policy	Area (ha)	City Quadrant	Num. sampling pt	Num. dog feces	Feces/ha/week	Feces/6 months	Total mass of feces (g)	Mass (g)/ha/week	Total mass (kg)/6 months
Fishcreek Provincial Park	On	1348	SE and SW	88	6	1.09	38,030.58	291.57	52.73	1848.18
Weaselhead	On	237	SW	24	0	0	0	0	0	0
Edworthy Park Lower	On	108	NW	12	1	1.33	3733.91	21.86	28.99	81.62
Southland low	On	37	SE	3	0	0	0	0	0	0
Stanley park	On	21	SW	5	0	0	0	0	0	0
Martindale	On	3	NE	2	0	0	0	0	0	0
Meadowlark	On	1	SW	2	0	0	0	0	0	0
West Hillhurst	On	1	NW	2	2	15.92	459.32	171.2	1362.37	39.32
Nose Hill Park	Mixed	1129	NW	51	3	0.94	27,481.21	56.74	17.71	519.77
Bowmont Park	Mixed	164	NW	25	6	3.82	407,182	359.64	228.95	976.26
Taradale	Mixed	22	NE	2	0	0	0	0	0	0
Southland park	Off	62	SE	12	21	27.85	44,897.62	654.16	867.61	1398.58
Edworthy Park Upper	Off	61	NW	8	9	17.9	28,266.82	397.89	791.58	1249.68
Riverpark	Off	21	SW	7	5	11.37	6207.04	117.42	266.97	145.77
Nosecreek	Off	18	NE	3	0	0	0	0	0	0
Inglewood Bird Sanctuary	No dog	36	SE	4	0	0	0	0	0	0
Inglewood Wildland	No dog	34	SE	9	0	0	0	0	0	0

In table are reported the number and mass of feces, average contamination rate per hectare per week, and the total feces estimated for the entire park in 6 months snow-free period.

**Figure 2:**
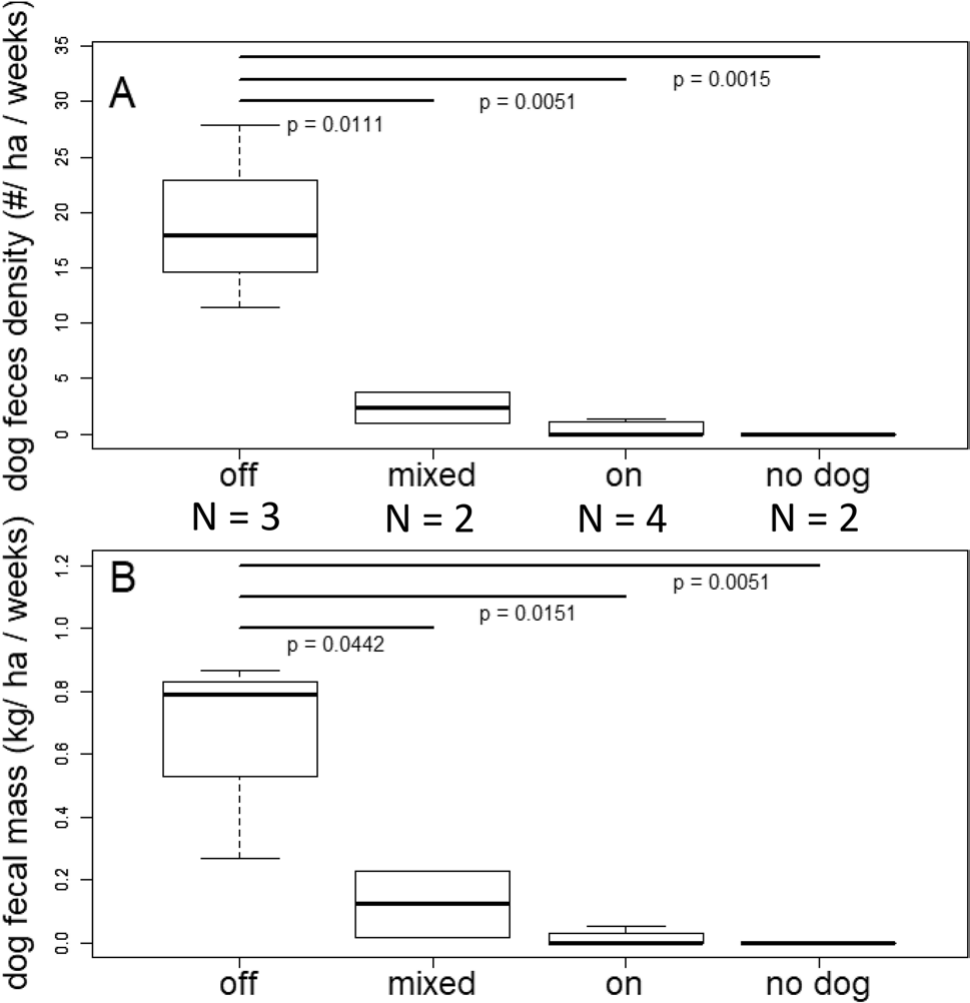
Dog fecal contamination expressed as the number (**A**) and mass in kg (**B**) in per hectare per week in the parks and natural areas in the City of Calgary (AB, Canada) surveyed in summer 2011 in relation to their dog leash policy: “off” for parks where dogs are allowed off-leash, “on” for parks where dogs are allowed on-leash, “mixed” for parks containing both off-leash and on-leash areas, and “no dog” for parks where dogs are not allowed. Both the numbers of feces and fecal masses show significant difference between off-leash parks and every other leash policy based on Tukey tests. The pairs that are significantly different are highlighted with horizontal lines and p-values.

On average, “off-leash” parks were more contaminated (Table 2). One-way ANOVA highlighted significant differences in density and amount of feces in these 4 types of parks (F_3,8_ = 14.02, p = 0.0015 for the number of feces; F_3,8_ = 9.45, p = 0.0053 for the mass of the feces). Tukey’s honest significant difference test multiple comparisons showed a significant difference in the number of feces between the “off-leash” and “mixed” pair (p = 0.0111), the “off-leash” and “on-leash” pair (p = 0.0015), and “off-leash” and “no-dog” pair (p = 0.0051; Fig. 2A, supplementary material Table S1). There were significant differences in the fecal mass between “off-leash” and “mixed” parks pair (p = 0.0442), “off-leash” and “on-leash” pair (p = 0.0048), and “off-leash” and “no-dog” pair (p = 0.0151; Fig. 2B, Supplementary material Table S2).

**Table 2:** Dog fecal contamination of urban parks of the City of Calgary assessed during a survey on random points within urban parks in the City of Calgary (AB, Canada) in the year 2011.

	Off-leash	Mixed	On-leash	No dogs
Number of feces (n/ha/week)	19.04 (± 2.77)	2.38 (± 1.02)	0.48 (± 0.13)	0
Fecal mass (g/ha/week)	642.05 (± 109.01)	123.33 (± 7.47)	16.35 (± 4.78)	0

Data were classified into 4 categories based on the parks’ leash policy. The fecal contaminations are reported as mean (± SE) number and mass (in gram) of feces found in each of the leash policy categories per hectare per week. “Off-leash” parks do not require dogs to be on leash, “On-leash” parks require dogs to be on the leash all the time, and “mixed” park has areas that are off-leash and on-leash. “No dogs” parks do not allow dogs at all.

Based on the GIS map of the areas managed by the City Parks and the off-leash areas, and the size of Fish Creek Provincial Parks, there are total of approximately 13,133 ha of park areas in the City of Calgary, including 1221 ha of the off-leash areas. Extrapolating the average of 642.10 g per ha per week in off-leash areas to the entire city, we estimate a total of 757.54 kg of fecal matter contaminating the off-leash areas every week during the snow-free period. Although the remaining 11,912 ha includes “no-dog” parks, given that “no-dog” areas are small and applying the average of 16.30 g per ha per week of “on-leash” areas, the remaining areas would have total of 665.79 kg of fecal matter every week, leading to 1423.33 kg of fecal matter every week for the whole city during the snow-free period.

### Dog feces distribution

**Figure 3:**
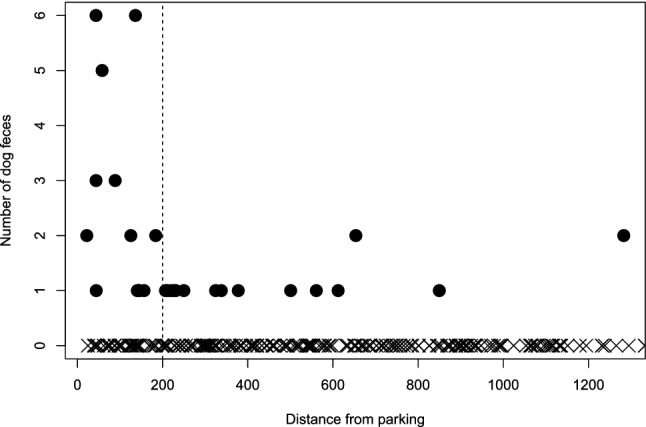
Distribution of dog feces found at each random plot by the distance from parking lots in the dog fecal contamination surveys carried out in 2011 in the city of Calgary (AB, Canada). Black dots indicate plots where some dog feces were found, X symbols indicate plot with no dog feces found. Dashed line indicates the threshold of 200 m from the nearest parking lot.

The variables that best explained the number of dog feces at each survey points were the distance of the point from parking lots, followed by the leash policy. Most plots with more than one dog feces were within 200 m from parking lots (Fig. 3). Most of the terrain and land cover variables resulted in little improvements to the model (see supplementary material S1). The final model included leash policy with a positive effect of off-leash policy and negative effect on on-leash policy on the number of dog feces relative to mixed leash policy parks, and negative effects of distance to parking (Table 3, also see supplementary material Figs. S2 and S3). The likelihood ratio of the final model to the null model was significant (*X*^*2*^ = 46.25, df = 3, p < 0.0001).

**Table 3:** Results of the Poisson model of the number of dog feces using data collected during a survey on random points within urban parks in the City of Calgary (AB, Canada) in the year 2011.

	Estimate	Std. error	Z value	Pr(>|z|)
Intercept	2.7761	0.9550	2.9069	0.0036
Park Leash Policy^a^: Leash off	1.332	0.4828	2.7589	0.0058
Park Leash Policy^a^: Leash on	− 1.3036	0.5710	− 2.2832	0.0224
Distance to parking	− 0.7859	0.1471	− 5.3446	< 0.0001

Park Leash Policy is a categorical variable indicating the leash policy of the park, ‘off’ is where dogs are allowed off-leash, and ‘on’ is where the dogs are allowed if they are on-leash; parks with both on-leash and off-leash areas (Mixed policy) were used as the base (reference) level; Distance to parking indicates the distance from the nearest parking lot in the parks.

^a^Reference category is Mixed leash policy.

## Discussion

In this study we aimed to understand the extent and spatial patterns of the fecal contamination in the urban parks of the City of Calgary for better management of the city parks. For the extent of the fecal contamination, according to our calculations, rate of fecal contamination of the public parks in the City of Calgary was close to 1.5 tons of dog feces per week. This amount of fecal material can be washed into rivers and streams by a heavy rain, contaminating the water downstream. Such a fecal contamination have not only serious direct effects on both public and wildlife health, but indirect effects through the increment of social conflict and an overall reduction of ‘wellness’ for people while in parks; the combination of these effects may cause significant loss to the “commons” of the city.

Parks with off-leash bylaws seemed to have significantly larger amount of dog feces left unpicked by owners, suggesting that dog owners in the City of Calgary usually are more likely to clean up after their dogs if the dogs are on leash. This might be due to the increased awareness of their dogs defecating if they are on leash, or because of decreased sense of being observed by other park users when their dogs are unleashed (i.e. defecating dog is not in immediate proximity of its owner, a likely occurrence in off-leash park) thus feeling less social pressure to clean up after their dogs; a known factor contributing to fecal contamination^43^. Particular concern for park users may be that more dog feces were found near parking lots, leading to more exposure to people and dogs. Although a parasitological study of dog feces in the Calgary found mostly non-zoonotic strains of *Giardia* spp. and *Cryptosporidium* spp.^44^, a recent study using the same dog samples found a highly virulent zoonotic parasite, *E. multilocularis*, at a prevalence of about 2.4%^45^. Because of its high fatality in humans, the presence of *E. multilocularis* is a cause of grave concern even at low prevalence^46^.

Our estimate of 19.04 dog feces per ha per week in off-leash parks in Calgary is within the range of dog fecal density reported from dog-friendly beaches in California where dogs were allowed off-leash, where the fecal densities were 2.1–4.9 dog feces per ha per day (14.7–34.3 per ha per week^47^). Perhaps there is a tolerance level of the density of dogs or dog feces for dog owners in North America above which they start avoiding that park. It would be interesting to know if urban parks in other cities have similar dog fecal densities or if there are city features that influence the fecal contamination. Unfortunately, while there are many studies on parasitological concerns from dog feces in public areas^23,48–52^, studies on the dog feces itself are limited.

Few studies that record densities of dog feces were difficult to compare. Rubel and Wisnivesky^19^ studied the dog fecal contamination in the suburbs of Buenos Aires, Argentina, where most dogs (pets and stray) were free ranging. They found 177 dog feces in 1939.5 m^2^ (912.6 feces per ha) in the parks in middle-income neighborhoods and 315 dog feces in 3284.6 m^2^ (959 feces per ha) in the parks in low-income neighborhoods, and even higher densities of feces on sidewalks, seemingly much higher than the urban parks of the city of Calgary. However, because this study did not control the time it took for those feces to accumulate, their results cannot be directly compared. In a study on Tibentan villages where dogs are often allowed to roam freely, Vaniscotte et al.^53^ found an average of 8.60 dog feces per ha, with peaks of 354 feces per ha. While their average is seemingly comparable to the fecal densities of the parks in Calgary, this study was not conducted in public parks, but in villages spaces, and again it did not control for the fecal accumulation time. Another study in France surveyed urban parks and rural settlements, but these surveys were conducted along the paths in the parks and settlements and not by areas^20^. Similarly, a study in Naples, Italy, surveyed along the transects placed throughout the city^21^.

While this study covered more parks than that of Rock et al.^1^, there are shortcomings. The socio-demographics of the neighborhoods surrounding each park were not assessed explicitly nor the activities of the park visitors studied. Part of the reason was that large parks such as Nosehill park and Fishcreek park straddle over several neighborhoods and are visited by people who do not resides in immediate neighborhoods of the parks, and are so large we could not make detailed observation of people. In fact, we had to omit small parks that were studied in Rock et al.^1^ as they were exceptionally small compared to the parks assessed in this study. However this study is rather complementary, assessing the patterns from different types of parks that provide important services to large number of people and dogs.

### Management implications in urban planning

Based on our data, defining and enforcing leash bylaws for the parks is likely an effective method for reducing fecal contamination. However, dog owners often find the ability to walk dogs off-leash to be an important quality of a park^54^, and their interests have to be balanced. Given the distribution of the feces within parks, to reduce the fecal contamination in public parks it is recommended to focus the efforts near parking lots and entrances, especially in off-leash parks. Designating areas near park entrances and parking lots as on-leash areas may have some effects. Studies by Jason and Zolik^55^ indicated education of dog-owners as effective strategies for reducing dog fecal contamination. Educating dog owners, especially of those who visit off-leash parks, on how to properly dispose feces and the harms they can cause may be a simple and effective approach. Posting signs at the parking lots and entrances of the parks reminding dog owners to clean up after their dogs may have impacts as well.

Considering the potential problems associated with the policy regarding the pet dogs and their use of public parks, conceptual framework of assessing and designing local government’s policies on pets were proposed^56^. In 2021, the Calgary’s City Council approved changes to the bylaw on pet ownership, to take effect beginning in January of 2022. Among several changes, the new bylaw defines the maximum number of dogs a person can supervise in an off-leash park at six, increase the penalties for acts of nuisances including dog feces, and authority for the city to ban a repeated offenders from off-leash parks. As these new policies take effects, it will be interesting to assess the effects on the dog fecal contamination in the Calgary’s public parks, using this study as a reference of the dog fecal contamination prior to the implementation of the new policies. We hope there will be similar studies in future with consistent methodologies conducted in different cities across the world, so that we may see patterns of fecal contamination over different cities, countries, continents or cultures. Such patterns can then be used to identify cultural characteristics or policies associated with fecal contamination, patterns that are potentially useful for policy makers.

## Supplementary Information

Supplementary Information.

## Data Availability

The data will be made available upon request to the corresponding author.

## References

[1] Rock Melanie J., Graham Taryn M., Massolo Alessandro, McCormack Gavin R. (2016). Dog-walking, dog-fouling and leashing policies in urban parks: Insights from a natural experiment designed as a longitudinal multiple-case study. Landsc. Urban Plan..

[2] Middle Isaac (2020). Between a dog and a green space: applying ecosystem services theory to explore the human benefits of off-the-leash dog parks. Landsc. Res..

[3] Frumkin Howard, Bratman Gregory N., Breslow Sara Jo, Cochran Bobby, Kahn Peter H., Lawler Joshua J., Levin Phillip S., Tandon Pooja S., Varanasi Usha, Wolf Kathleen L., Wood Spencer A. (2017). Nature contact and human health: A research agenda. Environ. Health Perspect..

[4] Toohey AM, Rock MJ (2011). Unleashing their potential: A critical realist scoping review of the influence of dogs on physical activity for dog-owners and non-owners. Int. J. Behav. Nutr. Phys. Act..

[5] Toohey A.M., McCormack G. R., Doyle-Baker P.K., Adams C.L., Rock M.J. (2013). Dog-walking and sense of community in neighborhoods: Implications for promoting regular physical activity in adults 50 years and older. Health Place.

[6] Christian H (2018). Encouraging dog walking for health promotion and disease prevention. Am. J. Lifestyle Med..

[7] Wood L (2017). Social capital and pet ownership—a tale of four cities. SSM-Popul. Health.

[8] Wood L, Giles-Corti B, Bulsara M (2005). The pet connection: Pets as a conduit for social capital?. Soc. Sci. Med..

[9] Weston MA (2014). Bark in the park: A review of domestic dogs in parks. Environ. Manage..

[10] Iojă CI (2011). Dog walkers’ vs other park visitors’ perceptions: The importance of planning sustainable urban parks in Bucharest. Romania. Landsc. Urban Plan..

[11] Teedon P (2014). Parental perceptions of the impacts the built environment has on young children׳s health: A qualitative examination and lay assessment amongst residents in four Scottish communities. Health Place.

[12] Derges J (2012). Complaints about dog faeces as a symbolic representation of incivility in London, UK: A qualitative study. Crit. Public Health.

[13] Robertson I (2000). The role of companion animals in the emergence of parasitic zoonoses. Int. J. Parasitol..

[14] Robertson ID, Thompson R (2002). Enteric parasitic zoonoses of domesticated dogs and cats. Microbes Infect..

[15] Garfield L, Walker M (2008). Microbial water quality and influences of fecal accumulation from a dog exercise area. J. Environ. Health.

[16] Hardin G (1968). The tragedy of the commons. Science.

[17] Matisoff D, Noonan D (2012). Managing contested greenspace: Neighborhood commons and the rise of dog parks. Int. J. Commons.

[18] Pearson C (2017). Combating canine ‘visiting cards’: Public hygiene and the management of dog mess in Paris since the 1920s. Soc. Hist. Med..

[19] Rubel D, Wisnivesky C (2005). Magnitude and distribution of canine fecal contamination and helminth eggs in two areas of different urban structure, Greater Buenos Aires Argentina. Vet. Parasitol..

[20] Knapp J (2018). Rural and urban distribution of wild and domestic carnivore stools in the context of Echinococcus multilocularis environmental exposure. Int. J. Parasitol..

[21] Rinaldi L (2006). Canine faecal contamination and parasitic risk in the city of Naples (southern Italy). BMC Vet. Res..

[22] Rinaldi L (2008). Giardia and Cryptosporidium in canine faecal samples contaminating an urban area. Res. Vet. Sci..

[23] Matsuo J, Nakashio S (2005). Prevalence of fecal contamination in sandpits in public parks in Sapporo City Japan. Vet. Parasitol..

[24] Smith A (2015). Urban park-related risks for Giardia spp. infection in dogs. Epidemiol. Infect..

[25] Mouton, M., & Rock, M.J. Débats autour des races canines et de la santé publique à Montréal et au Québec (2016–2019). *Can. J. Public Health* (2021). 10.17269/s41997-021-00550-3 PMC8825900 34382162

[26] Graham, T.M., *Montreal should look to Calgary for policies on pets*, in *The Suburban*. St Laurent. (2016).

[27] McCormack GR (2014). Physical activity patterns in urban neighbourhood parks: Insights from a multiple case study. BMC Public Health.

[28] Rock MJ (2016). Public engagement and community participation in governing urban parks: A case study in changing and implementing a policy addressing off-leash dogs. Crit. Public Health.

[29] Massolo A (2014). Echinococcus multilocularis in North America: the great unknown. Parasite.

[30] Calgary, T.C.o., *2019 Civic Census Results* (2019).

[31] The City of Calgary, *Biodiversity Report*. (2014).

[32] Rock M (2013). Pet bylaws and posthumanist health promotion: A case study of urban policy. Crit. Public Health.

[33] Halfpenny, J.C. *A field guide to mammal tracking in North America* (Big Earth Publishing, 1986).

[34] Liccioli S (2015). Feeding ecology informs parasite epidemiology: Prey selection modulates encounter rate with Echinococcus multilocularis in urban coyotes. PLoS ONE.

[35] Liccioli S (2012). Gastrointestinal parasites of coyotes (Canis latrans) in the metropolitan area of Calgary, Alberta Canada. Can. J. Zool..

[36] Liccioli S (2015). Assessing individual patterns of Echinococcus multilocularis infection in urban coyotes: non-invasive genetic sampling as an epidemiological tool. J. Appl. Ecol..

[37] Veit P (1995). Influence of environmental-factors on the infectivity of Echinococcus multilocularis eggs. Parasitology.

[38] Abdi, H., & Williams, L.J. *Tukey’s honestly significant difference (HSD) test.* Encyclopedia of Research Design, pp. 1–5 (Sage, Thousand Oaks, CA, 2010).

[39] Burnham, K.P., & Anderson, D.R.*Model Selection and Multi-Model Inference: A Practical Information-Theoretic Approach*. Secaucus (Springer, United States, 2002).

[40] Brooks ME (2017). glmmTMB balances speed and flexibility among packages for zero-inflated generalized linear mixed modeling. R J..

[41] Hartig, F. *DHARMa: residual diagnostics for hierarchical (multi-level/mixed) regression models*. R package version 0.4.3 2019 [cited 4; Available from: http://florianhartig.github.io/DHARMa/.

[42] Team, R.C., *R: A language and environment for statistical computing*. 2021, R Foundation for Statistical Computing: Vienna, Austraria.

[43] Lowe C (2014). Environmental and social impacts of domestic dog waste in the UK: Investigating barriers to behavioural change in dog walkers. Int. J. Environ. Waste Manag..

[44] Smith AF (2020). Molecular characterization of Giardia spp. and Cryptosporidium spp. from dogs and coyotes in an urban landscape suggests infrequent occurrence of zoonotic genotypes. Vet. Parasitol..

[45] Toews, E.A.W. Echinococcus multilocularis infections in domestic dogs. *Science* (2021). 10.1016/j.ijpara.2020.10.008 33482171

[46] Craig P (2003). Echinococcus multilocularis. Curr. Opin. Infect. Dis..

[47] Oates SC (2017). Daily relative dog abundance, fecal density, and loading rates on intensively and minimally managed dog-friendly beaches in central California. Mar. Pollut. Bull..

[48] Lykov, I., Pavlova, O., & Rudova, S. *Sanitary and hygienic aspects of urban environment pollution by dog feces*. in *IOP Conference Series: Earth and Environmental Science*. 2021. IOP Publishing.

[49] Cinquepalmi V (2013). Environmental contamination by dog’s faeces: A public health problem?. Int. J. Environ. Res. Public Health.

[50] Duncan KT (2020). Prevalence of intestinal parasites in fecal samples and estimation of parasite contamination from dog parks in central Oklahoma. Vet. Parasitol. Region. Stud. Rep..

[51] Zanzani SA (2014). Canine fecal contamination in a metropolitan area (Milan, North-Western Italy): Prevalence of intestinal parasites and evaluation of health risks. Sci. World J..

[52] Ristić M (2017). Epidemiological importance of green areas and public places contaminated with canine feces in urban environmental conditions. Acta Med. Med..

[53] Vaniscotte A (2011). Role of dog behaviour and environmental fecal contamination in transmission of Echinococcus multilocularis in Tibetan communities. Parasitology.

[54] Westgarth C, Christley RM, Christian HE (2014). How might we increase physical activity through dog walking? A comprehensive review of dog walking correlates. Int. J. Behav. Nutr. Phys. Act..

[55] Jason LA, Zolik ES (1981). Modifying dog litter in urban communities. Am. J. Public Health.

[56] Rock MJ (2014). Policies on pets for healthy cities: A conceptual framework. Health Promot. Int..

