# Supplementary material for: Fecal contamination of urban parks by domestic dogs and tragedy of the commons
Source: Sci Rep. 2023 Mar 1;13:3462. doi: 10.1038/s41598-023-30225-7 (PMC9977951; doi:10.1038/s41598-023-30225-7)
Supplement: Supplementary file 1 — Supplementary Information. [file 41598_2023_30225_MOESM1_ESM.docx]

# Supplementary material

#
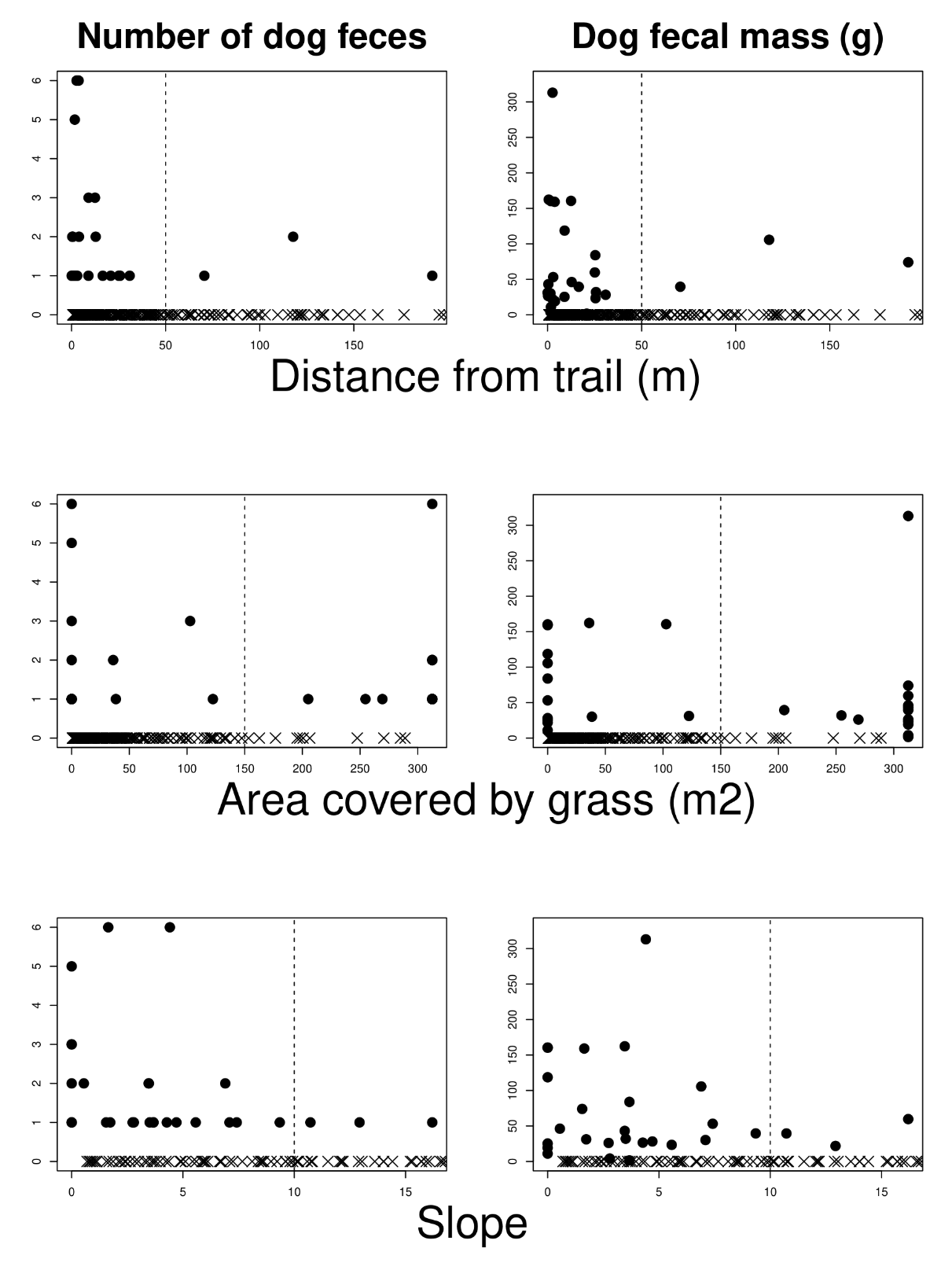


**Figure S1.** Distributions of dog fecal numbers (on left column) and dog fecal mass (on right column) found in the survey plots in the urban parks of the City of Calgary, in relation to the distance from trail in meters (top row), areas of the plot covered by grassland in square meters (middle row), and average slope of the plot (bottom row).


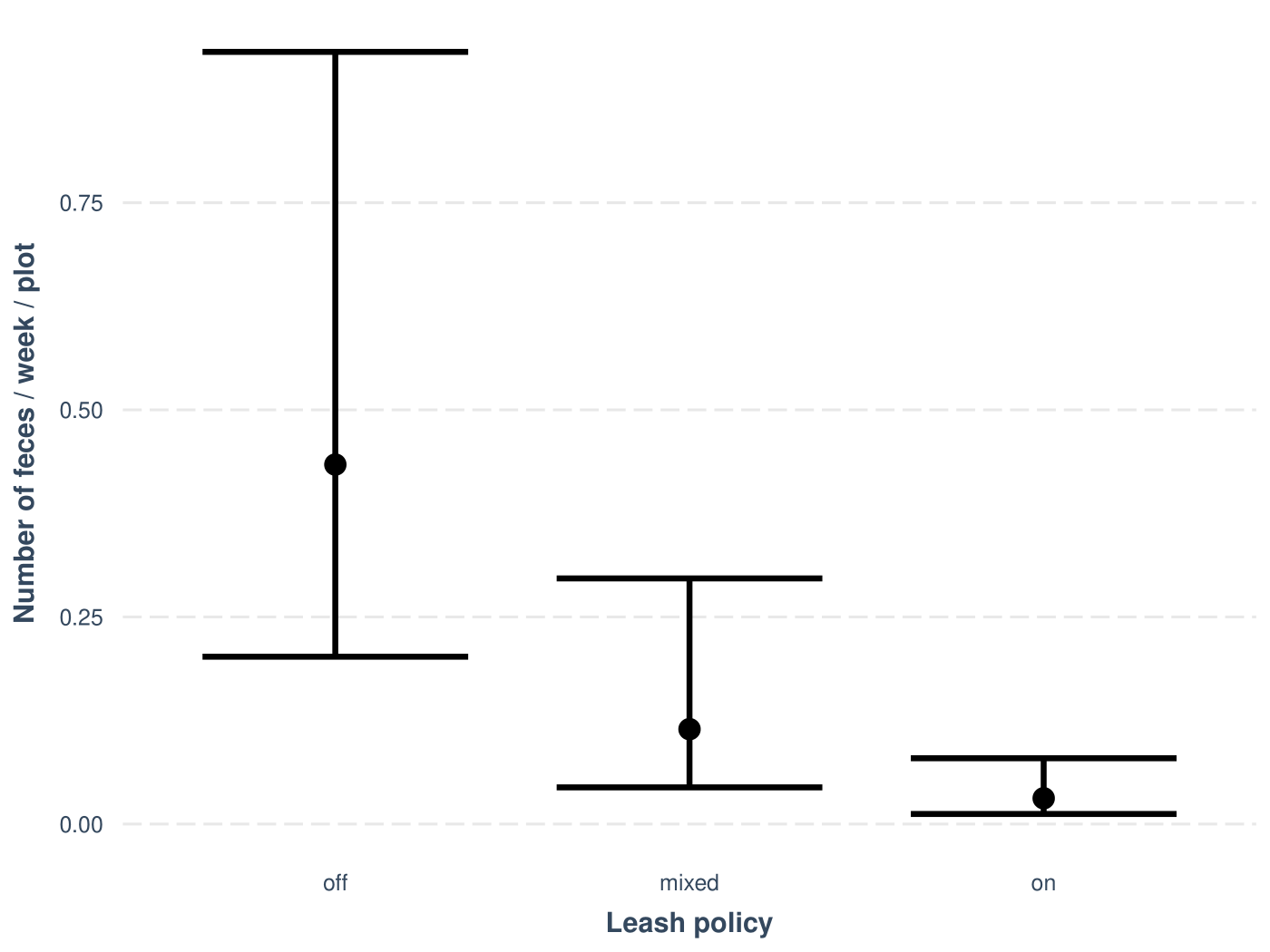


**Figure S2.** Predicted number of feces in a plot of 10 m radius in a week by the leash policy of the park based on the best performing model of the mixed effect Poisson distribution model among the urban parks in the City of Calgary, created using *jtools* package of R (version 2.1.3; Long, 2019). The predictions were made assuming the effects of other predictor variables are at their mean.


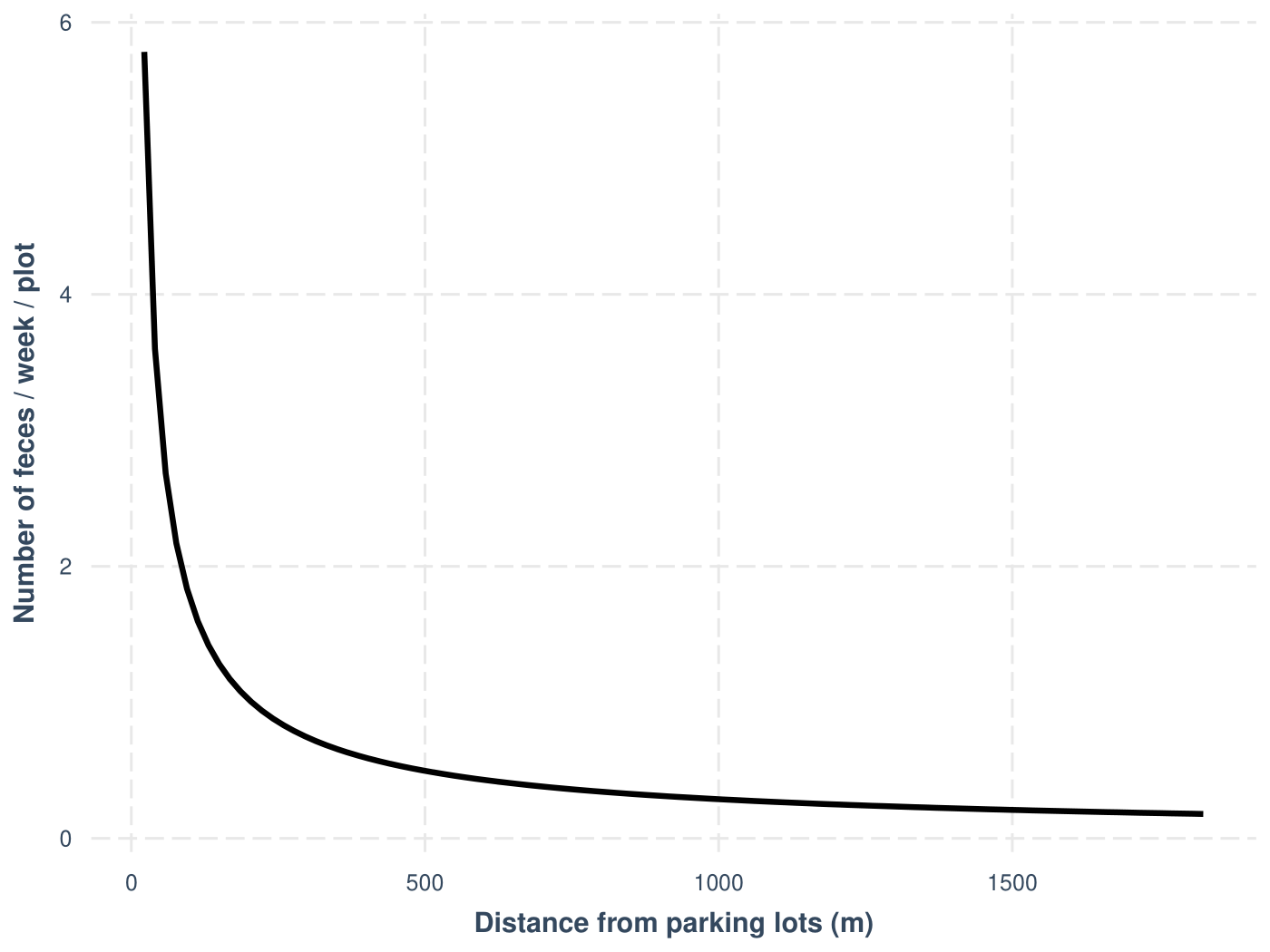


**Figure S3.** Predicted number of feces in a plot of 10 m radius in a week by the distance from parking lots based on the best performing model of the mixed effect Poisson distribution model in the urban parks in the City of Calgary, created using *jtools* package of R (version 2.1.3; Long, 2019). The prediction curve was plotted while assuming the effects of other predictor variables are at their mean.

**Table S1.** The results of Tukey’s honestly significant difference test of the number of dog feces found in the urban parks of the City of Calgary, sorted by the leash policy. *diff* column shows the difference in observed mean, *lwr* and *upr* columns shows the lower and upper end point of the interval respectively, and *p-adj* show the p-value after adjustment for the multiple comparisons. Note off-leash parks are significantly different in fecal mass with every other leash policies, but the differences between mixed, on-leash, and no-dog parks are not.

|  | *diff* | *lwr* | *upr* | *p-adj* |
| --- | --- | --- | --- | --- |
| no_dog-mixed | -2.3780 | -15.9516 | 11.1957 | 0.9409 |
| off-mixed | 16.6638 | 4.2728 | 29.0548 | 0.0111 |
| on-mixed | -1.8957 | -13.2522 | 9.4608 | 0.9481 |
| off-no_dog | 19.0418 | 6.6508 | 31.4327 | 0.0051 |
| on-no_dog | 0.4823 | -10.8742 | 11.8388 | 0.9990 |
| on-off | -18.5595 | -28.4722 | -8.6467 | 0.0015 |

**Table S2.** The results of Tukey’s honestly significant difference test of dog fecal mass found in the urban parks of the City of Calgary, sorted by the leash policy. *diff* column shows the difference in observed mean, *lwr* and *upr* columns shows the lower and upper end point of the interval respectively, and *p-adj* show the p-value after adjustment for the multiple comparisons. Note off-leash parks are significantly different in fecal mass with every other leash policies, but the differences between mixed, on-leash, and no-dog parks are not.

|  | *diff* | *lwr* | *upr* | *p-adj* |
| --- | --- | --- | --- | --- |
| no_dog-mixed | -0.1233 | -0.6763 | 0.4296 | 0.8887 |
| off-mixed | 0.5187 | 0.0140 | 1.0235 | 0.0442 |
| on-mixed | -0.1070 | -0.5696 | 0.3556 | 0.8781 |
| off-no_dog | 0.6421 | 0.1373 | 1.1468 | 0.0151 |
| on-no_dog | 0.0163 | -0.4463 | 0.4790 | 0.9994 |
| on-off | -0.6257 | -1.0295 | -0.2219 | 0.0048 |

**Table S3.** Comparison of mixed effects Poisson distribution model with the all the combination of variables considered. Full model includes variables of distance from parking (*parking*), distance from trail (*trail*), grassland land cover type (*grass*), average slope of the plot (*slope*), and leash policy of the park (*leash*) in addition to the random factor of parks. K is the number of parameters in the model, AICc is the corrected Akaike Information Criterion, Delta_AICc is the difference in the AICc from the best model, AICcWt is the Akaike weight that indicates the “weight of evidence” in support of that model among that candidate models, LL is the log likelihood of the model, and R^2^M and R^2^C shows the marginal and conditional pseudo-R^2^ using trigamma function calculated in “r.squaredGLMM” of R package *MuMIn* (version 1.43.17; Barton, 2009).

| **Model variables** | **K** | **AICc** | **Delta_AICc** | **AICcWt** | **LL** | **R^2^M** | **R^2^C** | |
| --- | --- | --- | --- | --- | --- | --- | --- | --- |
| 10. *parking leash* | 5 | 195.36 | 0 | 0.33 | -92.55 | 0.21 | 0.22 |  |
| 6. *slope parking leash* | 6 | 196.47 | 1.11 | 0.19 | -92.05 | 0.22 | 0.22 |  |
| 8. *grass parking leash* | 6 | 197.10 | 1.74 | 0.14 | -92.37 | 0.22 | 0.22 |  |
| 7. *trail parking leash* | 6 | 197.46 | 2.10 | 0.12 | -92.55 | 0.21 | 0.22 |  |
| 4. *slope grass parking leash* | 7 | 198.10 | 2.73 | 0.08 | -91.80 | 0.22 | 0.22 |  |
| 3. *slope trail parking leash* | 7 | 198.57 | 3.21 | 0.07 | -92.04 | 0.22 | 0.22 |  |
| 2. *grass trail parking leash* | 7 | 199.20 | 3.84 | 0.05 | -92.35 | 0.22 | 0.22 |  |
| 1. Full model | 8 | 200.21 | 4.84 | 0.03 | -91.78 | 0.22 | 0.22 |  |
| 12. *leash* | 4 | 221.17 | 25.81 | 0 | -106.50 | 0.16 | 0.16 |  |
| 11. *trail leash* | 5 | 222.76 | 27.40 | 0 | -106.25 | 0.16 | 0.17 |  |
| 9. *grass trail leash* | 6 | 224.79 | 29.43 | 0 | -106.21 | 0.16 | 0.17 |  |
| 5. *slope grass trail leash* | 7 | 226.41 | 31.04 | 0 | -105.96 | 0.16 | 0.17 |  |
| 13. Null model (park only) | 2 | 235.40 | 40.04 | 0 | -115.67 |  |  |  |

# cited references

Barton, K. (2009). MuMIn: multi-model inference. [*http://r-forge*](http://r-forge)*. r-project. org/projects/mumin/*.

Long, J. (2019). jtools: Analysis and presentation of social scientific data (R package version 2.0. 1).
